# An Unusual Case of Non-traumatic Chylothorax

**DOI:** 10.7759/cureus.32506

**Published:** 2022-12-14

**Authors:** Ariel Ruiz de Villa, Sanjae Spencer, Samantha Sircar, Raghav Bassi, Kipson Charles, Peters Okonoboh

**Affiliations:** 1 Internal Medicine, Hospital Corporation of America (HCA) & University of Central Florida (UCF) College of Medicine, North Florida Regional Medical Center, Gainesville, USA; 2 Internal Medicine - Critical Care, Hospital Corporation of America (HCA) & University of Central Florida (UCF) College of Medicine, North Florida Regional Medical Center, Gainesville, USA

**Keywords:** non traumatic, non-hodgkins lymphoma, thoracic duct injury, bilateral chylothorax, pleural empyema

## Abstract

Chylothorax refers to the presence of chyle in the paraaortic space. This entity most commonly occurs from injury to the thoracic duct, which carries chyle from the gastrointestinal tract to the bloodstream. Common etiologies around traumatic chylothorax include iatrogenic causes, such as surgical procedures near the thoracic duct and penetrating and blunt injuries to the chest. We present a case of a 49-year-old female who initially presented to the hospital with progressively worsening dyspnea leading to acute hypoxic respiratory failure requiring intubation and admission to the ICU. The patient’s presentation was initially thought to be due to and managed as an infectious process with empyema and septic shock until a diagnosis of nontraumatic chylothorax was established. In this article, we report a complicated case of chylothorax, initially masquerading as an infectious pulmonary process. We hope to raise this entity high on the differential when clinicians are confronted with the task of managing patients with similar presentations, which will, in turn, prevent delayed diagnosis and the unnecessary use of antibiotics.

## Introduction

Chyle is made up of multiple components not limited to chylomicrons, lymphocytes, triglycerides, and fat-soluble vitamins. The thoracic duct helps transport chyle from the intra-abdominal lymphatic system, which includes the lower extremity and retroperitoneal spaces up to the left subclavian and jugular veins where it then enters the bloodstream circulation. A well-known complication of injury to the thoracic duct is a chylothorax. This can lead to dyspnea as the chyle will behave and cause a similar presentation as a pleural effusion. When a chylothorax is present, it is often described in the literature as milky/white-appearing on thoracentesis/chest tube drainage; however, this milky/white-appearing fluid can be absent in up to 50% of patients with a chylothorax. For this reason, a high degree of suspicion is needed in order to diagnose a chylothorax if there is not an obvious traumatic injury. A literature review was conducted as we discussed our approach to care and recommendations for the management of patients with similar presentations.

## Case presentation

**Figure 1:**
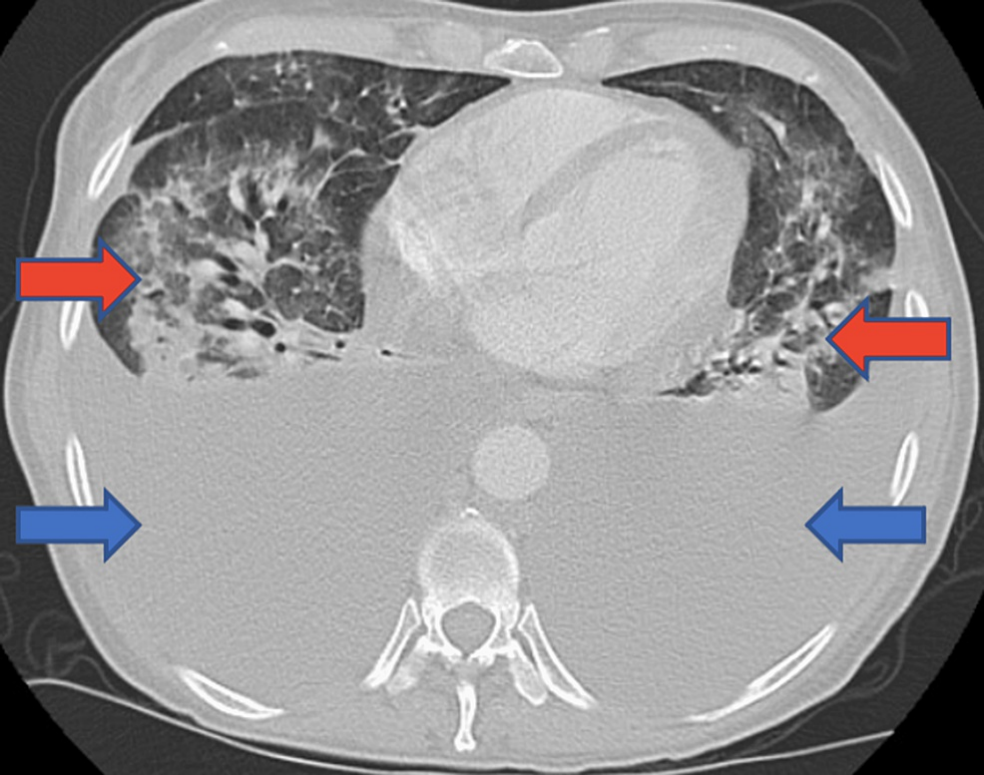
CT of the chest on the day of admission Blue arrows indicate large bilateral pleural effusions; Red arrows indicate severe bilateral pneumonia

Our patient was a 49-year-old female with a past medical history significant for stage IV non-Hodgkin’s lymphoma with recent chemotherapy administration, hepatitis B, and liver cirrhosis, and remote history of recreational drug use, who was admitted to the intensive care unit for acute hypoxic respiratory failure requiring intubation and mechanical ventilation. Before her presentation, the patient had progressive dyspnea due to community-acquired pneumonia, which was being treated in the outpatient setting. The patient’s respiratory compromise was severe, to the point that she could not ambulate or speak in full sentences. On examination, she was in obvious distress with a respiratory rate over 40 on bilevel positive airway pressure (BiPAP), 100% fraction of inspired oxygen (FiO2), and oxygen saturation (SpO2) of 87%. The patient was subsequently intubated. The X-ray done on admission was consistent with severe bilateral airspace disease and bilateral pleural effusions. CT of the chest showed similar findings of large bilateral pleural effusions with diffuse infiltrates hinting at an infectious process (Figure 1).

After intubation, the patient developed a right-sided pneumothorax. Bilateral chest tubes were placed for the evacuation of pleural fluid and relief of the pneumothorax. The pleural fluid was not sent for evaluation at this time. Shortly after, the patient developed distributive septic shock requiring multiple vasopressor agents. The patient was treated with a prolonged course of cefepime and vancomycin for community-acquired pneumonia progressing into sepsis. Admission labs are shown in Table 1.

**Table 1:** Pertinent laboratory data at the time of presentation AST=aspartate aminotransferase; ALT=alanine aminotransferase; MCV=mean corpuscular volume; BUN=blood urea nitrogen

Tests	Results	References	Units
White blood count	42.9	4.0 - 10.5	10³ uL
Hemoglobin	8.6	11.2 - 17.5	g/dL
Hematocrit	28.3	40.1 - 51	%
Platelets	123	150-400	10³ uL
MCV	100.3	79.0 - 92.2	fl
Sodium	140	136-145	mmol/L
Potassium	4.1	3.5-5.1	mmol/L
Chloride	108	98-107	mmol/L
Carbon dioxide	27.1	21-32	meq/L
Glucose	145	74-106	mg/dL
BUN	13	7-18	mg/dL
Creatinine	0.45	0.6-1.30	mg/dL
Albumin	2.76	3.4-5.0	g/dL
Total bilirubin	0.8	0.2-1.0	mg/dL
AST	43	15-37	units/L
ALT	31	13-56	units/L
Alkaline Phosphatase	220	45-117	units/L
Lactic acid	1.2	0.4-2.0	mmol/L

The patient’s chest tubes continued to drain, and pleural fluid samples were sent for analysis. Her respiratory status continued to improve, and she was successfully extubated on ICU Day 5. Pleural fluid analysis results are presented in Table 2, which are consistent with fluid clear/straw in color, no microbiological growth, and a fluid triglyceride level of 166 mg/dL. The patient was hemodynamically stable and transferred to the medical floor for continued broad-spectrum antibiotic treatment.

**Table 2:** Pleural fluid analysis at Days 5 and 15 from chest tube placements WBC=white blood cells; RBC=red blood cells; LDH=lactate dehydrogenase

Tests on Pleural Fluid	Day 5 Results	Day 15 Results	Units
Color	yellow	straw	subjective
Appearance	hazy	hazy	subjective
Fluid WBC	173	263	mm^3
Fluid RBC	2000	<2000	mm^3
Fluid Neutrophils	5	10	%
Fluid Lymphocytes	86	77	%
Fluid Eosinophils	1	1	%
Fluid Basophils	1	1	%
Fluid Glucose	127	127	mg/dL
Fluid Protein	2.3	1.3	g/dL
Fluid LDH	93	124	InternationalUnits/L
Fluid Cholesterol	<50	na	mg/dL
Fluid Triglycerides	121	166	mg/dL
Serum Total Protein	5.4	5.1	g/dL

Serial chest X-rays and physical examinations were evident for re-accumulation of pleural fluid bilaterally, with daily output from the chest tubes. Pleural fluid from the left side of the chest was sent again for analysis on Day 15 of admission. This analysis was consistent with an increase in fluid triglyceride levels at 121 mg/dL (Table 2). Per the pathologist's review, the pleural fluid did not show any malignant cells, only lymphocytes. Blood and pleural fluid cultures continued to be negative for any bacterial growth since admission. It was after this pleural fluid analysis that the diagnosis of re-accumulating chylothorax was made. As per the two-test rule commonly used to differentiate exudate vs transudate fluid, the effusions were both exudative with LDH being greater than 0.45 times the upper limit of the laboratory’s normal serum LDH. For our patient’s age and gender, this upper limit was 200 units/L. Light's criteria were not used due to incomplete laboratory values.

The patient was successfully treated with octreotide and a dietary restriction to medium-chain triglycerides. Approximately five days after starting treatment, there was a significant decrease in chest tube output. Chest tubes were removed, first the right side and a few days later, the left side, all while continuing octreotide and dietary adjustment. After 21 days of admission, the patient was discharged home with recommendations to follow up with a pulmonologist for additional management and radiological surveillance.

**Figure 2:**
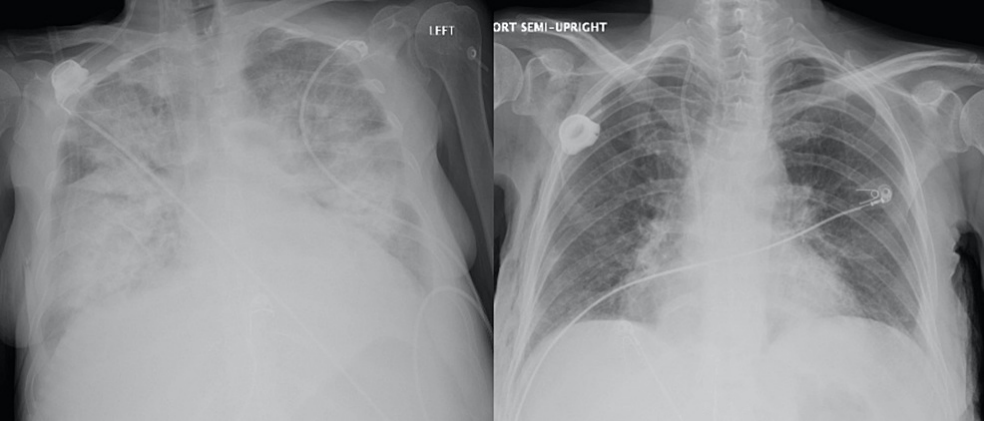
Chest X-rays Left: day of admission with severe bilateral pulmonary disease; Right: Day of discharge with the resolution of the disease

Chest X-rays (Figure 2) show a comparison and a marked improvement from the day of admission to the day of discharge.

## Discussion

Chylothorax is the accumulation of lymphatic fluid in the pleural space, causing pleural effusion. The lymphatic system in the body helps maintain fluid levels by absorbing excess fluid from the interstitial space and recirculating it back into the circulatory system. Chyle that is found in the lymphatic system contains triglycerides, T lymphocytes, electrolytes, proteins, immunoglobulins, and fat-soluble vitamins [1]. Thereby, it plays an essential role in fat absorption, immune response, and electrolyte homeostasis [2]. The thoracic duct is the major vessel that drains lymph from the entire body, except the upper right side of the body. The right upper limb, right breast, and right side of the head and neck are drained via the right lymphatic duct. The thoracic duct enters the mediastinum parallel to the aorta at the aortic hiatus in the diaphragm and travels upward to terminate at the left jugular and left subclavian veins. Given the trajectory the thoracic duct follows, any etiology that causes increased pressure in the chest that could block the lymphatic flow will eventually cause lymph fluid to leak into the pleural space, causing a chylothorax.

The most common cause of chylothorax is trauma. Whether iatrogenic (due to a surgical intervention), penetrating trauma, blunt force trauma, or forceful emesis, the thoracic duct is damaged, causing swelling and blockage of the duct with eventual rupture causing fluid to accumulate in the pleural space [1,3]. Non-traumatic etiologies of chylothorax include malignancy, superior vena cava thrombosis, sarcoidosis, benign tumors, and congenital anomalies. The most common non-traumatic cause is malignancy, accounting for 17%-46% of all cases [1,3]. Often, the presence of a chylothorax is the first presenting symptom of malignancy [4].

In our case, the patient was transferred to North Florida Regional Medical Center (NFRMC) from a nearby facility due to acute respiratory failure secondary to bilateral pleural effusions. She was subsequently intubated and bilateral chest tubes were placed. The patient had a known history of stage IV non-Hodgkin's lymphoma and had recently undergone chemotherapy with a regimen consisting of rituximab, cyclophosphamide, doxorubicin hydrochloride, vincristine, and prednisone (R-CHOP). Her last round of chemotherapy was two days prior to admission. CT chest showed a large periaortic upper abdominal soft tissue mass with associated ascites. Though there was no evidence of obstruction or leakage due to the lymphoma, the patient’s pleural fluid was consistent with chylothorax. There have been a few case studies hinting at the idea that chemotherapy, immunotherapy, and radiation for malignancies may cause chylothorax in the absence of obstruction [5-8].

A case study was done, looking at the prevalence of pleural effusions in patients with hematologic disease, including Hodgkin’s lymphoma, non-Hodgkin’s lymphoma, multiple myeloma, acute and chronic leukemia, and immunoglobulin G4 (IgG4)-related diseases. The most frequent hematologic disease to present with pleural effusions was non-Hodgkin’s lymphoma, with a prevalence of 35% [9,10]. Specifically, chylothorax is more common in non-Hodgkin’s lymphoma than any other type of hematologic disorder [11]. If left untreated, a parenchymal disease is very common and can present with mediastinal lymphadenopathy, usually associated with widespread disease. The cause of these pleural effusions may have similar etiologies as stated above but may also be due to direct pleural involvement by the tumor, obstruction via pulmonary and mediastinal lymph node enlargement, urinary tract compression, and reactive pleuritic or intrapleural extramedullary hematopoiesis [9,11]. Treatment of chylothorax is discussed below, but generally, treatment modalities are focused on eradication/management of the malignancy, usually by chemotherapy or radiation, and toward symptomatic relief of the chylothorax, including chest tubes, pleurodesis, and therapeutic thoracentesis. In patients with chylothorax secondary to underlying non-Hodgkin’s lymphoma, the prognosis is guarded, depending on the extent of the disease. Some pleural effusions have been shown to disappear in patients who have a good response to treatment of the underlying disease [12]. However, in many other case reports, the prognosis has been poor as the pulmonary and pleural involvement respond poorly to treatment [9,11,13,14]. 

In 84% of cases, the effusion occurs unilaterally, with 50% of them being on the right side of the chest [1]. Many patients will present with symptoms like other pleural effusions, such as dyspnea, chest pain, and cough, while others may be asymptomatic. But with slow leaking chylothorax, patients may present with electrolyte abnormalities, such as hyponatremia and hypocalcemia, and malnutrition with weight loss and muscle wasting. Chylothorax also predisposes patients to opportunistic infections, as the chyle contains immunoglobins and T lymphocytes [3]. CT chest is recommended for patients who have a nontraumatic chylothorax, as it can visualize a possible obstruction or malignant growth [1,15]. A newer imaging study, MRI lymphography can be used to visualize the course of the lymph through the lymphatic system by injection of contrast. This allows for direct visualization of a leak or obstruction of the lymphatic vessels [2]. But the final diagnosis is made from the pleural fluid analysis. The appearance of chylothorax pleural fluid may differ and cannot be used for diagnosis. Only about 20%-40% of chylothorax will have the traditional milky-white appearance [1]. Pseudochylothorax may also present with a milky appearance and lead to misdiagnosis. The definitive characteristic of chylothorax pleural fluid is the presence of chylomicrons or triglycerides > 110 mg/dL. Chylomicrons are complexes of proteins and lipids that are made in the jejunum and found in the thoracic duct after a meal, therefore being highly specific for a chylothorax. However, a pseudochylothorax pleural fluid analysis will have a milky appearance, pleural cholesterol > 200 mg/dL, pleural triglycerides < 110 mg/dL, and the presence of cholesterol crystals, all of which are due to a chronic inflammatory state and not due to injury to the thoracic duct [1,3].

There is no official management of chylothorax but depending on whether the cause is traumatic or non-traumatic, treatment can be surgical or conservative. For chylothorax that has a high output via chest tube (>1100 mL/24hr), usually traumatic, surgical intervention, such as thoracic duct ligation or embolization, is recommended [16]. For effusions that drain slowly, usually non-traumatic, medical management is preferred. This included dietary modifications and somatostatin analogs. Somatostatin analogs and octreotide reduce intestinal chyle production, thereby decreasing the volume of chyle traveling through the thoracic duct [1,3]. Our patient was managed with bilateral chest tubes, octreotide, and dietary modifications. Previously, chemical pleurodesis has been used as a treatment but has been shown to have minimal effect on chylothorax most likely due to the neutralizing effect of chyle [17].

Since malignancy should be on the top of the differential for nontraumatic effusions, treatment should be directed at identifying and treating the underlying disease. Our patient was referred to an outpatient pulmonary clinic for follow-up treatment with octreotide and advised to follow up with her oncologist within a week for continued treatment for her non-Hodgkin's lymphoma.

## Conclusions

Chylothorax is often described in the literature as a milky/white-appearing exudative effusion on a thoracentesis/chest tube drainage however this milky/white-appearing fluid can be absent in up to 50% of patients. As seen above, our patient had a clear/straw-colored effusion throughout her hospital course. The most common cause of a chylothorax is trauma either in the setting of iatrogenic causes due to surgical intervention, penetrating trauma, blunt force trauma, or forceful emesis. Non-traumatic etiologies of chylothorax include malignancy, superior vena cava thrombosis, sarcoidosis, benign tumors, and congenital anomalies with malignancy, accounting for 17%-46% of all cases. Clinicians should have a high suspicion of malignancies in patients presenting with chylothorax with non-Hodgkin's lymphoma being the most common culprit. Treatment of chylothorax consists of eradication/management of the malignancy, usually by chemotherapy or radiation, and toward symptomatic relief of the chylothorax, including chest tubes, pleurodesis, and therapeutic thoracentesis.
